# The applications of eHealth technologies in the management of asthma and allergic diseases

**DOI:** 10.1002/clt2.12061

**Published:** 2021-09-06

**Authors:** Alberto Alvarez‐Perea, Ves Dimov, Florin‐Dan Popescu, José Manuel Zubeldia

**Affiliations:** ^1^ Allergy Service Hospital General Universitario Gregorio Marañón Madrid Spain; ^2^ Gregorio Marañón Health Research Institute Madrid Spain; ^3^ Cleveland Clinic Florida FAU Charles E. Schmidt College of Medicine Weston Florida USA; ^4^ Department of Allergology ‘Nicolae Malaxa’ Clinical Hospital ‘Carol Davila’ University of Medicine and Pharmacy Bucharest Romania; ^5^ Biomedical Research Network on Rare Diseases (CIBERER)‐U761 Madrid Spain

**Keywords:** big data, eHealth, mHealth, social media, telemedicine

## Abstract

Portable devices, such as smartphones and mobile Internet access have become ubiquitous in the last decades. The term ‘eHealth’ stands for electronic health. The tools included in the eHealth concept utilize phones, computers and the Internet and related applications to improve the health care industry. Implementation of eHealth technologies has been documented for the management of different chronic diseases, including asthma and allergic conditions. Clinicians and patients have gained opportunity to communicate in new ways, which could be used cost‐effectively to improve disease control and quality of life of those affected. Additionally, these innovations bring new opportunities to academic researchers. For example, eHealth has allowed researchers to compile data points that were previously unavailable or difficult to access, and analyse them using novel tools, collectively described as ‘big data’. The role of eHealth become more important since early 2020, due to the physical distancing rules and the restrictions on mobility that have been applied worldwide as a response to the coronavirus disease 2019 pandemic. In this review, we summarize the most recent developments in various eHealth platforms and their relevance to the speciality of allergy and immunology, from the point of view of three major stakeholders: clinicians, patients and researchers.

## 1 INTRODUCTION

The term ‘eHealth’ is an abbreviation that stands for electronic health. The World Health Organization defines ‘eHealth’ as the use of information and communication technologies for health.^1^ However, there is not a universally accepted eHealth definition, and it is generally accepted to include an array of different technologies and tools, also known as health information technologies or medical informatics.^2^ eHealth utilizes computerized technologies, including the Internet and related applications, to improve the efficacy and efficiency of the health care industry.^3^ For a glossary of eHealth‐related terms, refer to Table 1.

**TABLE 1:** Glossary of eHealth tools and technologies

**App**: abbreviation for application software. Computer programme designed to perform a specific activity. They can run on computers, portable devices (mobile app) or web servers (web app).
**Artificial intelligence (AI)**: Simulation of processes associated with human intelligence by computers. It includes natural language processing, speech recognition or machine vision.
**Big data**: Recording and analysis of datasets that are too large, multidimensional, diverse and complex to be adequately processed by traditional software solutions.
**eHealth**: Abbreviation for electronic health. It includes a diverse group of tools that use computers and the internet to improve the efficacy and efficiency of the health care industry.
**Health informatics**: Integration of health care and computer science. It comprises electronic health records, computerized physician order entry, clinical decision support systems and software solutions for administrative tasks.
**mHealth**: Abbreviation for mobile health. Integration of mobile devices (e.g., smartphones) in the practice of health care.
**Natural language processing (NLP):** NLP is the ability of a computer programme to understand human language as it is spoken.
**Social media**: Interactive web‐based applications where users can share text posts, comments and multimedia files. They can be used to develop personal or professional social networks.
**Telemedicine**: Delivery of health‐related services performed via electronic information and telecommunication technologies. They can be real‐time interactive services or store‐and‐forward, which means acquiring data that is subsequently transmitted to a specialist for assessment.
**Wearable**: Hands‐free electronic device that can be worn as an accessory, clothing or as a part of the body (e.g., smart watches and bracelets and epidermal electronics resembling tattoos).

**FIGURE 1:**
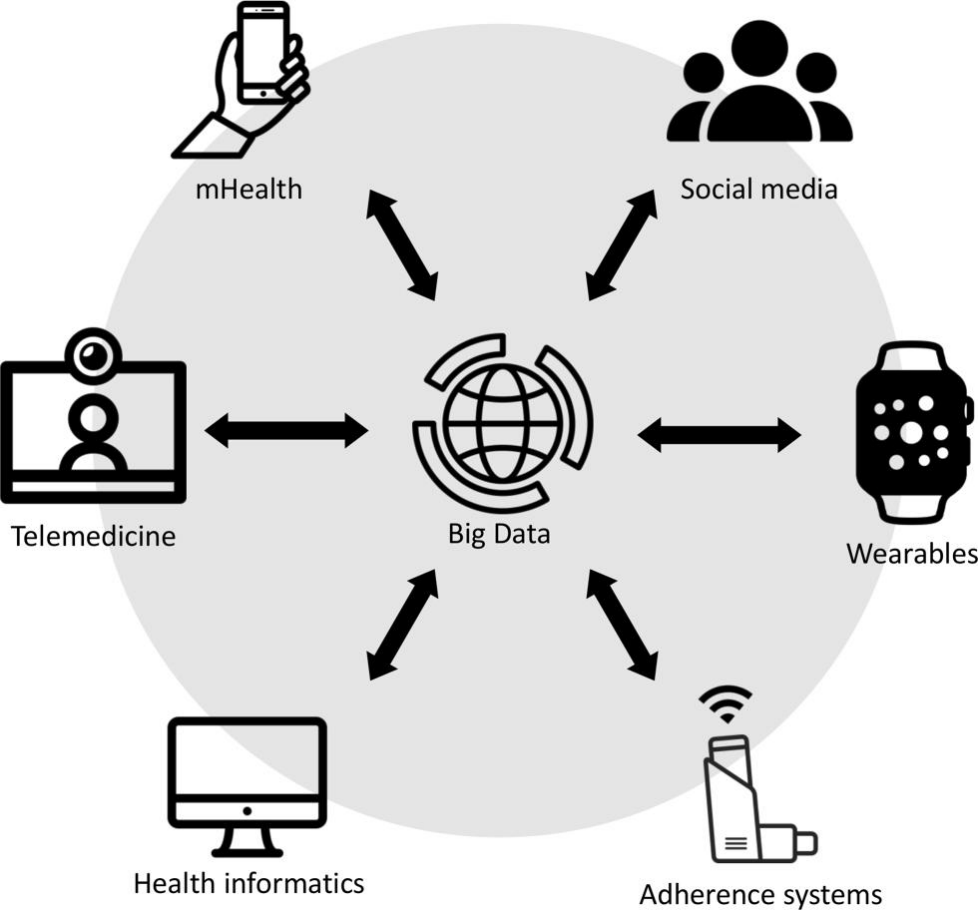
eHealth includes a variety of technologies that can be applied to the health care industry. The information generated by these tools can be used as a source of big data which, in turn, may improve the system

Among the technologies included under the eHealth umbrella are the use of mobile devices in medical care (mHealth), such as wearables, electronic diaries or adherence monitors; telemedicine, which is the adaptation of information and network technologies to the clinical practice and medical education; social media platforms and health informatics, such as electronic health records and clinical decision support systems. Finally, the analysis of information acquired through these tools can be performed using ‘big data’ technologies^4^ (Figure 1).

Adoption of new technologies in our personal lives has dramatically increased in the last few decades. Personal computers and laptops have led to lighter devices (e.g., smartphones or tablets). Mobile Internet access has become less expensive in developed countries and, therefore, widespread among the general population. In 2016, it was estimated that 75% of European households had a portable Internet connection.^5^

The healthcare environment has not been stranger to this tendency but technology adoption has proceeded at a slower pace compared to the personal use of devices and applications. Clinicians and patients have gained opportunity to communicate in new ways and compile data points that were previously unavailable, or very difficult to collect. Smartphones, ‘apps’ (applications) and other so‐called ‘smart’ devices, together with new analytic tools have opened new horizons for patients, clinicians and researchers.^6^ The coronavirus disease 2019 (COVID‐19) pandemic has been reported to dramatically increase the adoption rate of communication technologies in a few months’ time.^7^

The spectrum of allergic diseases comprises a variety of chronic conditions that can severely impair quality of life, including asthma, atopic dermatitis, allergic rhinitis and food allergy. These disorders have been reported to affect up to 20%–40% of the general population in developed countries. In addition, their prevalence is rising up to the point that it has described as an ‘allergy epidemic’.^8^ Allergic diseases require implementation of self‐management plans and patient empowerment. Unfortunately, this laudable goal may not be as easy achieved in real life clinical environments.^9^

Implementation of various eHealth technologies has already been documented for the management of different chronic diseases, including asthma and allergic conditions. However, there are many eHealth aspects that still remain uncertain and under investigation. In most cases, study evidence is still limited, lower quality, based on the current research standards or inconsistent.^10^ It is still necessary to identify and define eHealth interventions or combinations of them that may be adequate for different patients and allergic disorders. As with any clinical intervention, some eHealth tools may have a negative impact on certain vulnerable patient subpopulations.^11^ In addition, privacy remains an important concern that must always be addressed with the highest standards of protection, as mandated by the specific government regulations.^12^

In this review, we summarize the most recent developments in various eHealth platforms and their relevance to the speciality of allergy and immunology, from the point of view of three major stakeholders: clinicians, patients and researchers.

## 2 eHEALTH FOR THE ALLERGY CLINICIAN

### 2.1 mHealth

Web applications (apps), wearables and other personal monitoring devices have a potential to improve the management of asthma and allergic conditions. Proposed benefits of asthma apps include the ability to longitudinally collect symptoms and inhaler usage data, thus allowing the detection of changes over time to help patients and caregivers determine whether the symptoms are worsening. Data from external information sources, including weather, allergen load and air quality reports, can be integrated.^13^ The collected data from the apps can be shared with the allergy clinicians during clinic visits or via telemedicine portals.

An online health diary platform can be used to assess the effects of personal behaviour and environmental exposure on allergic rhinitis and asthma symptoms. A pilot study of an online asthma diary included 132 patients, who provided 25,016 diary entries. The analysis of this data showed the effects of multiple risk factors for asthma symptoms, including contact with persons with influenza‐like illness, perception of cold or hot temperature, high‐intensity exercise, dehumidifiers at home, second‐hand smoke, poor sleep quality, poor quality of indoor and outdoor air.^14^ Adolescents and their caregivers agreed with the acceptability of using smartphones for real‐time asthma monitoring.^15^ An app‐based portable spirometer has recently been found to be comparable to a conventional spirometer.^16^ The ability to access high quality lung function measures outside of the clinical setting might potentially aid patients in recognition of changes in asthma status over time. The data points collected from this kind of platforms could lead to a more complete evaluation of the symptoms and their triggers by the allergy clinicians that would extend beyond the time‐limited interactions with patients during a clinic or telemedicine visit.

**FIGURE 2:**
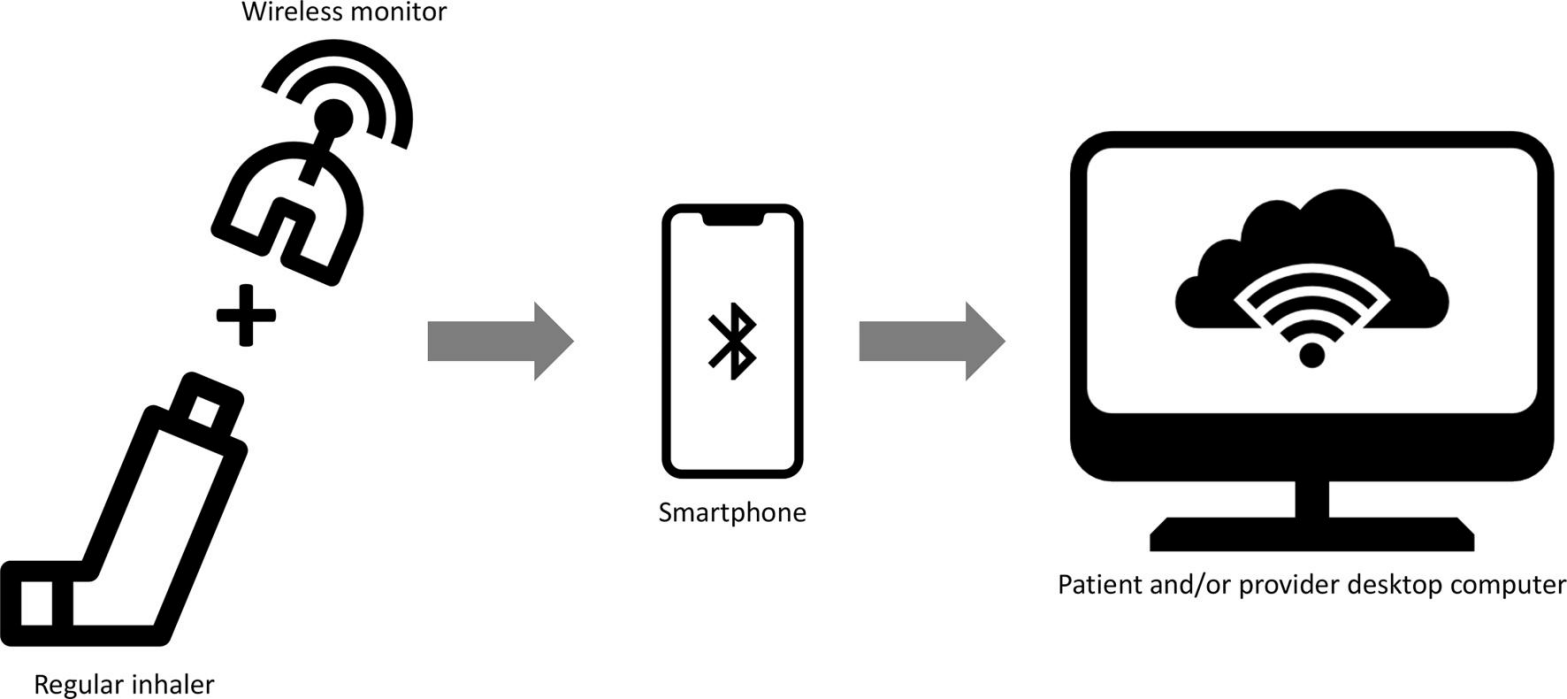
Some electronic inhaler sensors can be applied to a regular inhaler. Others are already built into the device. They collect adherence data and can submit it wirelessly to a portable device, usually a smartphone or tablet, which can upload it to a cloud‐based service, so it can be processed from a desktop computer, either by patients or healthcare providers

Electronic inhaler sensors can track the time, frequency and location of short‐acting β‐agonist (SABA) use. A pilot study of a sensor‐driven asthma management platform, which included 120 patients, reported a significant reduction in SABA use, increased number of symptom‐free day, and improvements in asthma control during a 30‐day period^17^ (Figure 2).

### 2.2 Electronic health records

Electronic health record systems have been widely adopted in health care, thus generating large amounts of real‐world data. Natural language processing techniques is an artificial intelligence (AI) approach that extracts information from clinical narratives in electronic health records.^18^ In addition, electronic health records allow temporal condition pattern mining that may reveal previously unknown associations between diagnoses, thus informing future research into causation.^19^ Applications of these technologies to the allergy practice are still in early developmental stages. There are studies that have calculated food allergy or drug allergy prevalence in different populations mining electronic health records.^20^, ^21^, ^22^ A natural language processing algorithm has demonstrated to be able to ascertain asthma analysing such records.^23^ Potential future uses of AI might include detection patients who need allergy care before their diseases deteriorate. This could be particularly relevant to address the case of patients that missed their consultations or interrupted their treatments due to the COVID‐19 pandemic.

### 2.3 Telemedicine

Telemedicine is rapidly gaining acceptance as a convenient way of accessing medical services worldwide. In 2019 alone, the Cleveland Clinic, a large health system in the United States, completed 41,000 virtual visits, including all medical specialities. Six months into 2020, the volume of virtual visits jumped to more than 500,000.^24^ There are studies that support that telemedicine is as effective as in‐person visits for outpatient treatment of asthma.^25^ A study including 169 children with asthma compared disease control and patients satisfaction after 6 months of in‐person visits or telemedicine care and found them to be similar.^26^ There are also surveys that have found that both patients and allergists tend to be as satisfied with telemedicine, or even prefer it over in‐person visits.^25^, ^27^ As a result of the COVID‐19 pandemic, telemedicine has become an integral part of allergy and immunology clinics daily practice all over the world.^28^ Telemedicine could be especially useful for providing proper speciality care for allergic conditions in rural communities, where in‐person visits are problematic, due to shortage of resources.^29^ In countries, such as the USA, where the private practice is common, there are still significant policy barriers, including a fragmented reimbursement system, that challenge more widespread implementation.^30^ In the near future, when combined with device sensor input, electronic health records and big data AI, telemedicine is expected to deliver transformational changes in the way allergy care is delivered.

### 2.4 Social media

An online social network is defined as an app or website that enables users to communicate with each other. The professional organizations in the field of allergy and immunology use social networks for member relations, public advocacy and education and Twitter and Facebook are actively utilized to stay up‐to‐date with the latest news.^31^

Twitter use by individual allergy practitioners often peaks during the annual meetings of the major professional organizations, such as the European Academy of Allergy and Clinical Immunology (EAACI), the American College of Allergy, Asthma, and Immunology (ACAAI), the American Academy of Allergy, Asthma, and Immunology (AAAAI) and the World Allergy Organization (WAO).^32^, ^33^ Table 2 provides a list of social network pages of the most relevant professional organizations in the field of allergy and clinical immunology. Tweets are tagged with a meeting‐specific searchable hashtag. Research reports from the AAAAI and the Spanish Society of Allergology and Clinical Immunology (SEAIC) meetings have demonstrated the each meeting hashtag activity surpasses several million impressions.^32^, ^33^ Twitter chats are used to engage the general public and patients by individual allergists, and professional and patient‐support organizations. At the time of this writing, social networks, such as Twitter, are being used widely for real‐time information sharing and collaboration among the international health care community during the COVID‐19 pandemic.^34^

**TABLE 2:** Social network pages of the most relevant professional organizations in the field of allergy and clinical immunology

American Academy of Allergy, Asthma, and Immunology (AAAAI):
Twitter: https://twitter.com/AAAAI_org
Facebook: http://www.facebook.com/AmericanAcademyofAllergyAsthmaandImmunology
Instagram: https://www.instagram.com/aaaai_org/
YouTube: https://www.youtube.com/channel/UCWto9iPcGxRn3s2AyzpwQHw
American College of Allergy, Asthma, and Immunology (ACAAI):
Twitter: https://twitter.com/ACAAI
Facebook: https://www.facebook.com/TheACAAI
YouTube: http://www.youtube.com/user/allergists
European Academy of Allergy and Clinical Immunology (EAACI):
Twitter: https://twitter.com/EAACI_HQ
Facebook: https://www.facebook.com/EAACI
Instagram: http://instagram.com/eaaci
YouTube: http://www.youtube.com/user/EAACIHeadquarters
Latin American Society of Allergy and Immunology (SLAAI):
Twitter: https://twitter.com/SocLatAlergia
Facebook: https://www.facebook.com/groups/slaai/
YouTube: https://www.youtube.com/channel/UCQipYqjC1CkO19ohZJ‐pU9A
World Allergy Organization (WAO):
Twitter: https://www.twitter.com/worldallergy
Facebook: https://www.facebook.com/worldallergyorg
Instagram: https://www.instagram.com/worldallergy
YouTube: https://www.youtube.com/user/TheWOChannel

Twitter Journal Clubs offer an alternative to the traditional journal club. Allergy and immunology was the first medical speciality to have a dedicated journal club on Twitter in 2008,^35^ and this concept was taken to new heights of online reach and participation in 2021 by the monthly Allergy & Immunology Journal Club (https://twitter.com/aimededjc). A blogging platform gives more control to the author in terms of website design, structure and web address versus social media posts.^35^ Randomized studies have shown that posting on social media about a specific scientific article can increase its metric statistics, such as the number of readers^36^ and citations^37^ and impact factor.^38^

With more than one billion monthly users, YouTube is the second largest search engine, after the flagship service provided by its parent company, Alphabet/Google. However, what patients find on YouTube can be misleading and it was only occasionally correct.^39^, ^40^, ^41^, ^42^ Allergy clinicians and professional society organizations can provide accessible and accurate information utilizing social media channels and YouTube. eHealth has a great potential for professional education, for example, Conferences On‐Line Allergy (COLA) is a lecture programme by ACAAI (childrensmercy.org/cola_online). Its YouTube channel with more than 400 videos and 3200 subscribers has received over 490,000 views as of 2021.

The risks to social media use by the allergy clinicians are related to breaches of patient confidentiality, professionalism, privacy, malpractice and liability.^43^ Personal attacks and harassment of physicians have also been reported.^44^ Table 3 provides recommendations for allergists considering having a presence on social media.

**TABLE 3:** Recommendations aimed at maintaining a professional social media presence

• Social media is a method of building relationships and a professional reputation. Content posted may have a positive or negative effect among patients and colleagues.
• Identify yourself and be cautious of your online image. Adhere to institutional rules and recommendations.
• Distinguish private from professional online presence by using different accounts and adjusting privacy settings. Redirect patients’ requests to professional accounts. Avoid providing specific medical advice.
• Maintain patients’ confidentiality. Avoid disclosing specific information. It can still be linked to the particular patient, even after anonymization. Do not acknowledge a physician‐patient relationship online.
• Once content is online, it is almost impossible to remove and can quickly spread beyond one’s control.
• Social media abusers can be blocked and reported to the platform where the issue was raised.

## 3 eHEALTH FOR PATIENTS AND PATIENT REPRESENTATIVES

eHealth interventions are recognized as an effective way to promote patient engagement and health care access in chronic diseases, such as diabetes.^45^, ^46^ However, eHealth utilization has been described to be low among patients with asthma^47^ and evidence supporting clinical benefit is still preliminary. Research protocols have found specific benefits of eHealth application in patients with allergic conditions.^4^

### 3.1 mHealth

In the field of mHealth, studies conducted in patients with asthma have demonstrated an improvement in symptoms and a reduction in the number of visits to the emergency room, by using home spirometers and a SMS alert service.^48^, ^49^ A randomized trial in children with asthma using a monitoring mobile app showed a decrease in urgent care visits, although it failed to prove any difference in emergency room visits or hospitalizations.^50^

Regarding the use of mHealth for remote monitoring, a recent pilot study demonstrated that a smartphone built‐in microphone, used in combination with a cloud‐based platform could approximate pulmonary function tests.^51^ This kind of applications could open new possibilities for more convenient and remote evaluation of these patients.

Therapy adherence remains a challenge in the management of patients with chronic conditions.^52^ Specifically, patients with asthma frequently fail to use their controller medications.^53^ In many cases, patients with anaphylaxis, despite their life‐threatening disease, are reluctant to carry their epinephrine auto‐injectors or to use them.^54^ mHealth has been proposed as a possible solution to these challenging clinical situations.^55^ Reminder systems and gamification of the management, including digital and real‐life rewards, may seem like obvious choices.^56^ However, a recent meta‐analysis found important limitations regarding the evidence supporting the potential effects of mHealth in asthma control. They found that these interventions have only reported modestly improvement of inhaler adherence and reduction of rescue inhaler use, without an actual impact on asthma control.^57^ In addition, their long‐term effects remain to be evaluated.^48^

Uncontrolled asthma is associated with extensive use of healthcare resources. In a significant number of cases, this lack of control is related to inadequate inhalation technique and adherence to treatment.^58^ In recent years, the interest in the field of inhaler adherence has increased steadily, supported by mHealth apps and wearables. Most of the currently available studies have addressed electronic reminders and inhaler tracker interventions^59^ (Figure 2). A recent observational study described a 14.5% increase in inhalers adherence by using digital trackers among patients with asthma and COPD during the COVID‐19 pandemic.^60^ An electronic monitoring sensor attached to inhalers has proven to increase asthma control in paediatric patients, as well as caregivers quality of life.^61^ A multi‐centre observational study concluded that a gamification strategy using a mobile app could be useful to monitor adherence to asthma medication.^62^ Additionally, patients’ feedback on these devices has also been described to be positive.^63^

MASK‐Air is a mobile app that has been designed for patients with allergic rhinitis, allergic conjunctivitis and asthma. It is part of Allergic Rhinitis and its Impact on Asthma (ARIA) and it has been translated into multiple languages.^64^ Through this app, patients are able to record their daily symptoms using analogue visual scales. Subsequently, they can download reports and share them with their healthcare providers in order to optimize the management of their diseases. Its utility has been thoroughly validated for multiple purposes, both for patients and professionals.^65^ In addition, global data is available to researchers, which is leading to a better understanding of respiratory allergic diseases.^66^

mHealth has also been used to provide allergic patients with pollen counts in order to predict symptoms^67^ and improve self‐control.^68^ A pilot study led in Australia found that most participants found this service useful.^69^ However, an evaluation of different apps containing pollen forecasts found that their quality was in need of improvement.^70^

A recently published open‐label randomized controlled trial demonstrated that an epinephrine auto‐injector smart case, attached to a mobile app, reduced anxiety in patients with anaphylaxis. In addition, it improved the patient’s perception of the management of an acute episode of anaphylaxis.^71^

There are several devices under development that aim to detect traces of food allergens in prepared meals. There is a patent for a device using molecularly imprinted polymers,^72^ which would detect peanuts, tree nuts, fish, shellfish, wheat, eggs, milk and soy.^73^ At the time of this writing, there is no published evidence supporting such claims.

Heterogeneity in the quality of the studies endorsing the use of mHealth in allergic diseases is high and the number of robust randomized controlled trials is still low.^10^ Information contained in most of the apps about asthma available for iOS and Android‐based smartphones do not contain evidence‐based recommendations. Thus, their actual clinical efficacy is doubtful.^74^

### 3.2 Telemedicine

Telemedicine has several potential benefits for allergic patients, including better access to health care and cost reduction.^75^ A 2014 study found that e‐visits could save as much as $5 billion worldwide.^76^ A 2018 study concluded that telemedicine can be used to remove penicillin allergy suspicion (‘delabelling’), saving the patients time and monetary expenses (over $30,000), and it was associated with high satisfaction.^77^ Asthma studies have demonstrated that internet‐based teleconsultation systems or face‐to‐face real‐time telemedicine were comparable to in‐person visits.^26^

Telemedicine received a tremendous boost in acceptance and utilization during the COVID‐19 pandemic.^78^ The most heavily affected countries saw their allergy practices rapidly being converted to telemedicine within days.^79^ In many cases, the radical change in practice had to happen without much of the needed preparation or advanced planning.^80^ Despite this almost overnight transformation, there are reports that allergic patients' satisfaction with telemedicine during the pandemic was high. A recent study conducted in a Spanish allergy unit, demonstrated that half of the patients that underwent a telephone consultation during the first peak of the pandemic would welcome this practice after the resolution of the epidemiological emergency.^81^

### 3.3 Social media

Social media has become a popular source of health information for the asthma and allergy patients and the general population. A survey conducted by the European Commission in 2014 revealed that 59% of the respondents searched the Internet for health‐related information, furthermore, 17% did so on social media.^82^ In Spain in 2015, another official survey found that 37.6% used social media in a similar fashion.^83^ An observational study in a Spanish allergy unit reported that half of the patients included in the study searched the Internet for Allergy information.^84^ A study, based on a survey among caretakers of food‐allergic patients attending a Spanish Paediatric Allergy Unit, described that over two thirds of them used social media and 25% gathered information related to their disease. The most popular food allergy‐related use of social media was receiving food allergen information, followed by medical information and socializing with other patients.^85^

Moreover, patients show a significant level of engagement during the annual meetings of the major professional organizations. It has been described that, during the period from 2013 to 2016, up to 12.3% of the users of the official hashtags of the SEAIC annual congresses were patients.^33^

The main limitation for the use of social media for patients is reliability. There is no standardized method to measure the characteristics of information available in social networks.^86^ Quality of the information contained in videos posted on YouTube has been found to be low for asthma, rhinitis, immunodeficiencies^35^ and food allergy.^42^ Similar findings were shown in research of photos posted on Instagram, Twitter and Facebook about asthma inhalers.^87^ On a positive note, a recent randomized controlled trial suggested that personal Twitter posts by physicians are effectively changing attitudes of the general public regarding COVID‐19 activity restrictions.^88^ The use of Internet and social media has been proposed as a valuable resource for the long‐term management of patients with asthma and anaphylaxis.^89^

Safeguarding privacy in eHealth services from the patients’ perspective is of the utmost importance. The use of eHealth services should not diminish the quality of services or the social interaction required in health care. Additionally, eHealth service providers and professionals need to act to maintain and improve access, data accuracy and security regarding information storage.^90^

## 4 eHEALTH FOR ACADEMIC AND CLINICAL RESEARCH

### 4.1 mHealth

mHealth technologies have a great potential for research into asthma and allergic conditions, however published studies are still limited.^10^

There is a multitude of mHealth apps for smartphones and tablets, with information on risk factors for allergic rhinitis and asthma, including pollen and air pollution data. However, there is evidence that the quality and reliability of the information contained on most of these apps is low. Consequently, the number of apps providing adequate scientific information is small.^91^ Several apps incorporated clinical data with self‐monitoring, patient’s feedback and education and empowerment tools. A collaborative and uniform approach is required for epidemiology, genetics and pathogenesis research.^68^ mHealth apps allow rapid collection of data from patients with asthma and allergic conditions in real‐life, thus increasing the studies size at a relatively low cost compared to clinical trials. By daily monitoring with geolocation tools, the apps might facilitate the development of management programmes for allergic diseases aggravated by environmental factors. mHealth technologies are already in use for electronic diaries in clinical trials of allergen immunotherapy, they make monitoring of large groups of patients feasible, allowing cost‐effective real‐life interventional studies, while data on pollen and fungal spore concentrations determine exposure‐symptom thresholds. mHealth applications in food and venom allergy need further investigation.^10^

Adherence apps, including reminder systems, sometimes with rewarding and feedback components, might have a potential to alleviate nonadherence in clinical trials. Researchers have the responsibility to detect inconsistencies in mHealth apps and wearables.^4^ Electronic sensors, attached to inhaled asthma medications, monitor the date, time and frequency of their use, with data transmission to encrypted servers. Data are accessible to the research team through smartphone apps.^92^, ^93^ Wearable devices, such as watches or bracelets, may be used with specific sensors in a non‐intrusive manner.^94^, ^95^ Due to the rapid development of wearables and smartphones, Internet of Things‐enabled technology will impact future clinical research.^96^

### 4.2 Electronic health records

In the field of health informatics, electronic health records are used in a wide range of clinical trials, including for improvements of the patient‐clinician interface and data analysis. The low adoption rate of standardized procedures and protocols for electronic health records platforms may lead to difficulties in coordinating research activities.^97^ Electronic health records analysis use for drug hypersensitivity warrants further investigation.^98^ Computerized physician order entry and mHealth facilitate real‐time identification of potential study subjects for clinical research.^99^

Clinical decision support system currently developed to optimize allergic rhinitis control, combined with innovative tools, such as MACVIA‐ARIA Sentinel NetworK for allergic rhinitis (MASK‐rhinitis), make possible innovative clinical trials.^100^, ^101^, ^102^

### 4.3 Telemedicine

Telemedicine use the European Union has been reported as cost‐effective in several trials.^103^ Patient enrolment limitations have been associated with unplanned time extension and early termination of clinical research trials.^104^ A recent research survey explored the utilization of telemedicine‐based resources. Respondents largely agreed that telemedicine was an important research approach, and regarded multimedia content, capabilities of mobile devices and three‐way calls as important enhancements. Research opportunities included consenting participants at their homes or remote clinic sites, telesurveys, file transfer, screenshare, video call recording and integration of medical devices.^105^ Furthermore, exploration of telemedicine in asthma and allergy practice through large studies is warranted.^106^

### 4.4 Social media and infodemiology

Social media services are increasingly used to obtain or share scientific content, including curated information from clinical trials, and to utilize the available communities for crowdfunding and crowdsourcing.^32^, ^33^, ^107^, ^108^ Interested allergists may use social media to discuss research ideas, future and ongoing research projects.^31^, ^35^

The term ‘infodemiology’ has been used to define the use of content publicly available in electronic mediums, most frequently the Internet, to inform public health. Tools, such as Google Trends may reflect a real‐life epidemiological approach to allergic rhinitis. A study found that searching Google Trends for a certain set of keywords (‘hay fever’, ‘allergy’ and ‘pollen’) could detect seasonality of allergic rhinitis in different European countries.^109^ Twitter microblogging platform has been used in health research in multiple trials.^110^ Geolocation techniques and the networks of users allow gathering spatial information.^111^ In a Japanese study, data on pollen counts and the number of tweets during an allergic rhinitis season were analysed using Granger causality.^112^ A UK research of social sensing for allergic rhinoconjunctivitis evaluated Twitter data and generated a dynamic spatial map of pollen levels based on user reports of symptoms with great similarity to measurements from the pollen monitoring stations.^113^ An Australian study validated the practical application of state‐of‐art Deep Learning in the context of pollen allergy surveillance on Twitter.^114^ Natural language processing (NLP)^115^ was used in a US study which explored large public text‐based social media data from Twitter, Facebook and Reddit. The data were analysed using a patented algorithm that applied NLP to online free‐text discussions in order to detect adverse events related to allergen immunotherapy.^116^ Reddit, with its application programming interface, has become a widely studied social media platform for understanding opinions on health issues, especially among young people.^117^

Image‐based social media platforms, especially Instagram and Pinterest, deserve further research assessment,^118^ while at the same time posing additional ethical issues.^119^, ^120^ Pinterest, with its image searching lens, may be analysed for visual and textual medical‐related information.^121^ Furthermore, social media research on allergy information‐seeking behaviour is also needed.^122^

### 4.5 Big data

The application of big data to allergy and immunology research generates complex, multidimensional and massive quantities of information.^68^, ^123^, ^124^ A prediction model using big data to assess asthma emergency department visits had a good accuracy.^125^ Big data can be obtained from genome, transcriptome, epigenome, microbiome and metabolome studies. Recently it was published a study protocol to estimate the prevalence of severe asthma using big data methods.^126^ Integration of big data from large cohorts, using machine learning approaches, is also needed to identify disease biomarkers and define molecular phenotypes/endotypes required for precision medicine approaches.^127^ Personal health profiles captured by individuals through mHealth technology may add another big data dimension to clinical trials with deeper phenotyping and real‐time exposome profiling.^128^

## 5 CONCLUSIONS

Adoption of new technologies has increased dramatically in the recent years including in the healthcare setting. Digital health technologies, generally positively perceived by patients and clinicians, reveal substantial promise for disease monitoring and personalization of treatment.^129^ Future trials investigating technology‐based interventions should include standardized patient‐reported outcomes as endpoints to better clarify their positive impact on patient education, environmental trigger control, symptom monitoring, comorbidities management and medication adherence.^130^

Allergy and immunology clinicians, patients/patient representatives and researchers have all benefited directly or indirectly from the recent developments in electronic health (Table 4).

**TABLE 4:** Practical applications of eHealth for different members of the Allergy community

	Feature	Application
Clinicians	mHealth	Mobile apps used as electronic diaries in asthma and rhinitis, leading to more detailed symptom monitoring.
	Adherence monitors	Provide reliable information on the use of prescribed treatments, creating an opportunity to support patient education.
	Electronic health records	Increased opportunities to mine clinical data in order to get a more realistic analysis of activity. Clinical decision support systems might be an opportunity to provide better care to patients, reinforcing evidence‐based recommendations.
	Telemedicine	Extend the reach of the practitioner beyond the limitations of a physical clinic.
	Social media	Real‐time information‐sharing with colleagues, engaging the general public, enabling novel methods of patient education, accessing professional education.
Patients	mHealth	Electronic diaries and gamification may increase awareness of symptoms and improve control. Reminder systems may improve treatment adherence.
	Adherence monitors	Reinforce medication use, leading to better control of the diseases and improvements in the quality of life of patients and caregivers.
	Wearables	Provide real‐time information to improve the perception of the disease and early detection of triggers.
	Telemedicine	Give better access to health care resources regardless of geographical location and cost reduction.
	Social media	Source of medical information and education. Engagement with healthcare professionals and other patients.
Researchers	mHealth	Mobile apps and sensors may be cost‐effective methods of rapid and valid collection of data.
	Adherence monitors	Increase adherence to clinical trials interventions, giving information about the causes of non‐adherence.
	Wearables	Automatic collection of data about subjects and activities.
	Electronic health records	Data mining can be used to identify potential research participants.
	Social media	Source of easy to collect, real‐time data. Increase visibility and impact of publications.
	Big data	Process huge datasets, including subjects profiling, phenotyping, exposome analysis, etc.

A wide‐range of eHealth applications are available to allergy practitioners. Electronic data diaries and wearables, such as device sensors, health informatics and social media, mHealth terminals, apps and online health diary platforms may help allergists to closely monitor clinical symptoms and adherence. Monitoring devices can detect trigger factors of rhinoconjunctivitis and asthma exacerbations, such as pollen counts or air pollution. Cloud‐based diary platforms allow a real‐time follow‐up of symptoms and spirometer values, thus facilitating digital management interventions. During the COVID‐19 pandemic, the use of telemedicine has proven successful in assisting patients with asthma. The growing participation of specialists in social networks has led to their evolution as useful tools for physician and patient education. Twitter utilization has resulted in several million impressions during the allergy societies professional meetings, with Twitter journal clubs further increasing the number of readers and citations of specific scientific articles.

Patients and patient representatives may also benefit from mHealth. Recent data have shown positive trends in asthma monitoring, control and therapy adherence. Inhaler tracker interventions and reminder trials in asthma are ongoing. The detection of traces of food allergens in prepared meals is being explored. However, due to the paucity of randomized controlled trials and heterogeneity of the studies, not enough evidence has been generated to validate the clinical efficacy of many of the eHealth tools. Telemedicine has demonstrated real life benefits, facilitating access to specialists and decreasing costs, when compared to face‐to‐face visits. Social media are not only a source of medical information for the general population but a new instrument for patients that allows them to take an active part in the meetings of professional organizations. All the above contrasts with some limitations as safeguarding the privacy and the reliability of the shared information.

mHealth also offers great potential for allergy and immunology research, mostly in the fields of clinical trials and real‐life investigations. Several apps for smartphones and tablets are available for pollen counting and air pollution level measurements.

mHealth allows efficient and cost‐effective data collection via electronic diaries and geolocation tools which are particularly well‐suited for allergen‐specific immunotherapy research. Wearable devices and electronic sensors attached to inhalers offer additional research opportunities. Data validation and privacy concerns require additional academic and legislative efforts to address the present risks and inconsistencies. In the field of health informatics, the implementation of electronic health records for clinical trials has greatly facilitated data gathering and analysis, and helped to improve patient‐clinician interface and communication. Clinical decision support systems may be based on big data and AI. Using these tools might potentially improve, not only clinical care, but also the design and implementation of innovative clinical trials. Nevertheless, the lack of standardized procedures across systems and sites limits research coordination and opportunities. Telemedicine has shown to be cost‐effective in clinical trials, it was well‐received by patients and clinicians alike, and with ‘teleconsent’ facilitated enrolment in clinical trials. The role of social media in discussing research ideas through Twitter microblogging and the image‐based social media platforms, such as Instagram or Pinterest, has been widely appreciated by both patients and healthcare providers. Finally, data mining of electronic health records holds a great promise and the field is in rapid development. High expectations are placed on improving prediction models, systems biology and personal health profiles. As a recurring theme, ethical issues, privacy and security of information remain the major concerns looking into the future.

The dramatic innovations in healthcare technologies during the last few decades bring unique opportunities to improve effectiveness and enhance wellness and quality of life in a cost‐effective way across the spectrum of healthcare delivery and academic research. The validation of the above‐mentioned eHealth tools and strong privacy protections are the key factors for long‐term positive impact.

## CONFLICTS OF INTEREST

The authors declare that they have no conflicts of interests regarding this article.

## AUTHOR CONTRIBUTIONS

Alberto Alvarez‐Perea participated in the design of the review and drafted the manuscript. Ves Dimov and Florin‐Dan Popescu drafted the manuscript and revised it critically. José Manuel Zubeldia participated in the design of the review, drafted the manuscript and revised it critically. All authors read and approved the final manuscript.
